# Extracellular vesicles from subjects with COPD modulate cancer initiating cells phenotype through HIF-1α shuttling

**DOI:** 10.1038/s41419-023-06212-1

**Published:** 2023-10-14

**Authors:** Ilaria Petraroia, Patrizia Ghidotti, Giulia Bertolini, Francesca Pontis, Luca Roz, Melissa Balsamo, Paola Suatoni, Ugo Pastorino, Anna Maria Ferretti, Gabriella Sozzi, Orazio Fortunato

**Affiliations:** 1 Epigenomics and biomarkers of solid tumors, Fondazione IRCCS Istituto Nazionale dei Tumori, Milano, Italy; 2 Thoracic Surgery Unit, Fondazione IRCCS Istituto Nazionale dei Tumori, Milano, Italy; 3 Istituto di Scienze e Tecnologie Chimiche-CNR, Milano, Italy

**Keywords:** Cancer stem cells, Non-small-cell lung cancer

## Abstract

Chronic obstructive pulmonary disease (COPD) is a risk factor for lung cancer development. COPD induces activation of hypoxia-induced signaling, causing remodeling of surrounding microenvironmental cells also modulating the release and cargo of their extracellular vesicles (EVs). We aimed to evaluate the potential role of circulating EVs from COPD subjects in lung cancer onset. Plasma**-**EVs were isolated by ultracentrifugation from heavy smoker volunteers with (COPD-EVs) or without (heavy smoker-EVs, HS-EV) COPD and characterized following MISEV guidelines. Immortalized human bronchial epithelial cells (CDK4, hTERT-HBEC3-KT), genetically modified with different oncogenic alterations commonly found in lung cancer (sh-p53, KRAS^V12^), were used to test plasma-EVs pro-tumorigenic activity in vitro. COPD-EVs mainly derived from immune and endothelial cells. COPD-EVs selectively increased the subset of CD133^+^CXCR4^+^ metastasis initiating cells (MICs) in HBEC-sh-p53-KRAS^V12high^ cells and stimulated 3D growth, migration/invasion, and acquisition of mesenchymal traits. These effects were not observed in HBEC cells bearing single oncogenic mutation (sh-p53 or KRASV12). Mechanistically, hypoxia-inducible factor 1-alpha (HIF-1α) transferred from COPD-EVs triggers CXCR4 pathway activation that in turn mediates MICs expansion and acquisition of pro-tumorigenic effects. Indeed, HIF-1α inhibition or CXCR4 silencing prevented the acquisition of malignant traits induced by COPD-EVs alone. Hypoxia recapitulates the effects observed with COPD-EVs in HBEC-sh-p53-KRAS^V12high^ cells. Notably, higher levels of HIF-1α were observed in EVs from COPD subjects who subsequently developed cancer compared to those who remained cancer-free. Our findings support a role of COPD-EVs to promote the expansion of MICs in premalignant epithelial cells through HIF-1α-CXCR4 axis activation thereby potentially sustaining lung cancer progression.

## Introduction

Cigarette smoking can be considered the main risk factor for the development of chronic obstructive pulmonary disease (COPD) and for the onset of lung cancer [1]. However, only 20% of smokers develop COPD [2], whereas the incidence of lung cancer is higher in subjects with COPD [3]. Since COPD and lung cancer display the activation of some common pathways related to inflammation or growth factors [4], it is likely that the presence of COPD could be a potent driver for lung cancer progression. In NSCLC, we and other shown that CD133 represents a robust marker to identify cells endowed with stem like properties and cancer initiating potential (cancer-initiating cells-CICs) [5–7]. It is now well-recognized that CICs represent an heterogeneous population comprised of cells deputed to specific function, endowed with different properties and characterized by specific markers [8]. In lung cancer for instance different markers have been described to possibly identify CIC subset, suggesting a great phenotypic heterogeneity of such population [9–12]. We also provided evidence for functional heterogeneity of lung CICs, demonstrating that CD133^+^ CICs co-expressing the chemokine receptor CXCR4 possess increased disseminating and mesenchymal properties and are defined metastasis initiating cells (MICs) [13]. Epithelial to Mesenchymal Transition (EMT) is an important mechanism for the acquisition of migration and pro-metastatic traits and it is also associated with the induction of MICs phenotype [13].

The pronounced inflammation in lung microenvironment of COPD subjects, caused by the recruitment of several immune cells such as neutrophils [14] and macrophages [15], represents the major source of reactive nitrogen and oxygen species that lead to cell DNA damage. Furthermore, it has been shown that the reduced air flow in COPD subjects stimulated the formation of an hypoxic microenvironment in the lungs [16]. In particular, the activation of hypoxia-inducible factor 1-alpha (HIF-1α) signaling pathway has been associated with a reduction in lung functionality [17]. Notably, HIF-1α has also been described to regulate self-renewal of CICs, supporting their maintenance and cancer progression [18] and also directly regulates CXCR4 expression [19].

An hypoxic condition has been shown to increase the extracellular vesicles (EVs) production in several lung inflammatory diseases [20] and cancers [21, 22]. EVs are a heterogeneous group of membrane-limited particles produced by almost all cell types. Since these vesicles transfer bioactive molecules (proteins, lipids, and nucleic acids), they have been recognized as key mediators of cell-to-cell communication. Indeed, growing evidence suggested that alterations in extracellular vesicles production are closely associated with chronic airway inflammatory diseases like COPD [23, 24]. In particular, a high number of microvesicles released by platelets and immune cells was observed in the plasma of COPD subjects [25] and the pro-inflammatory content of EVs secreted by monocytes induced the destruction of alveolar wall [26]. Interestingly, endothelial cells contribute to the release of CD31^+^-EVs that stimulate COPD progression by inducing apoptosis in the neighboring endothelial cells [27]. Furthermore, epithelial cells damaged by cigarette smoking release a high number of EVs that promote inflammation through IL-8 and VEGF [28].

Although COPD and lung cancer share common features, the processes behind the development of lung cancer in COPD subjects are yet to be understood, in particular regarding the potential role of circulating EVs in promoting the progression of premalignant epithelial lesions.

In this study, we characterized circulating EVs isolated from heavy smoker volunteers with or without COPD to investigate their ability to induce pro-tumorigenic features in different subtypes of bronchial epithelial cells and in the subset of CICs. We analyzed the effect of hypoxia on EVs release and the role of the transcription factor HIF-1α in the induction of protumorigenic phenotype caused by COPD-EVs. Finally, we showed an increased amount of HIF-1α inside EVs from COPD subjects who have developed lung cancer.

## Results

### Isolation and characterization of EVs from heavy smoker volunteers and COPD subjects

**Fig. 1:**
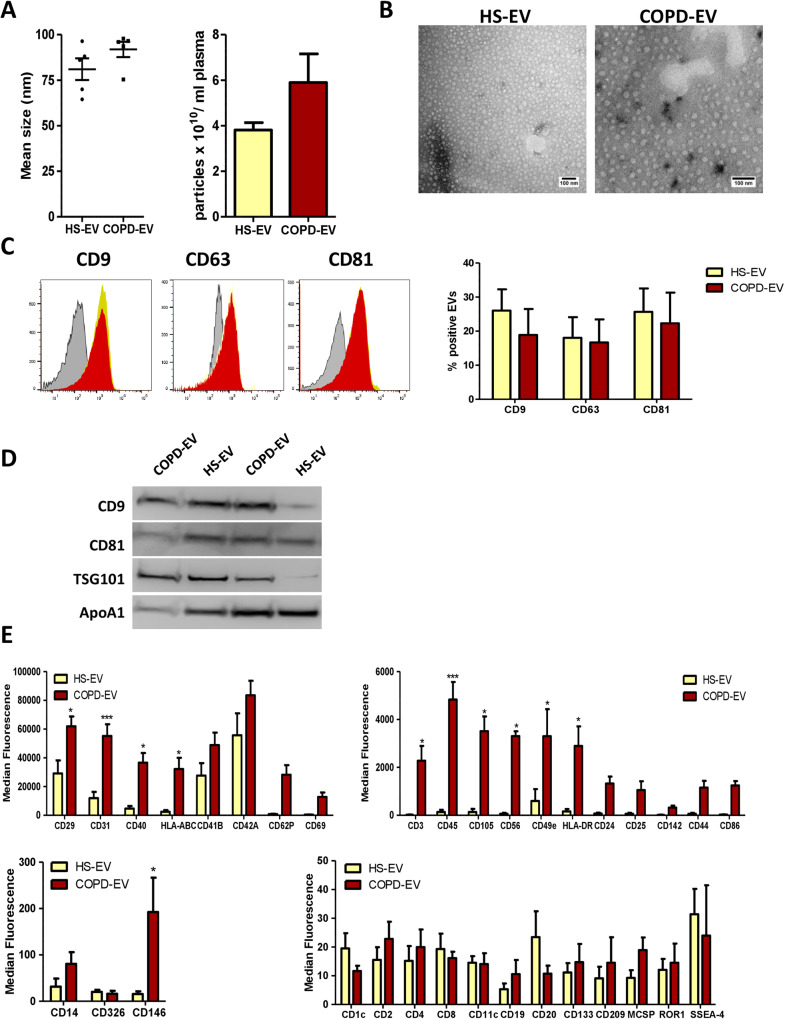
Characterization of plasma EVs. **A** Concentration and size distribution of plasma-derived EVs from subjects with (COPD-EVs) or without (HS-EVs) COPD using nanoparticle tracking analysis (*n* = 5 per group). **B** Representative TEM images showing the spherical morphology and size distribution of plasma-derived EVs. **C** Flow cytometry analysis of conventional EV markers on COPD- and HS-EVs showing the presence conventional markers (CD9, CD81, CD63) (*n* = 3 per group). **D** Western blot of EVs from COPD and HS volunteers for EV markers (CD9, CD81, Tsg101) and a marker of lipoprotein contamination (ApoA1). **E** Profiles of cell surface markers of plasma-derived EVs determined by flow cytometry. The values are expressed as median fluorescence intensity (*n* = 5 per group) *p < 0.05, ***p < 0.001 versus HS-EVs. Data are expressed as mean and SEM.

EVs from heavy-smoker volunteers (HS-EVs) and from COPD subjects (COPD-EVs) were isolated from 1 ml of plasma and characterized in accordance with Minimal Information for Studies of Extracellular Vesicles (MISEV) guidelines [29]. Nanoparticle tracking analysis showed that HS-EVs and COPD-EVs had a mean size of 81.02 nm (SEM = 5.945 nm) and 91.88 nm (SEM = 4.176 nm), respectively, without any significant differences between the two groups and a similar total number of EVs was counted in the plasma (Fig. 1A). Plasma EVs displayed a spherical shape and a relatively wide size distribution by TEM (Fig. 1B). Flow cytometry analysis detected the presence of canonical EVs markers such as tetraspanins CD9, CD63 and CD81 on their surface with comparable levels between HS-EVs and COPD-EVs groups (Fig. 1C). Western blotting further confirmed the presence of EVs’ markers and the presence of lipoprotein contaminants in our preparation mainly due to the isolation method adopted and the complexity of the starting biofluid (Fig. 1D).

To identify the cellular origin of plasma EVs, we performed a profiling analysis of lineage-specific markers using the MACSPlex Kit. Interestingly, we observed that COPD-EVs had higher levels of endothelial markers such as CD31, CD105, and CD146 than HS-EVs (Fig. 1E). Moreover, the higher abundance of CD3, CD45, HLA type I and II markers on EVs of COPD subjects suggested a derivation from immune cells.

### COPD-EVs increase the number of CD133^+^CXCR4^+^ cells

**Fig. 2:**
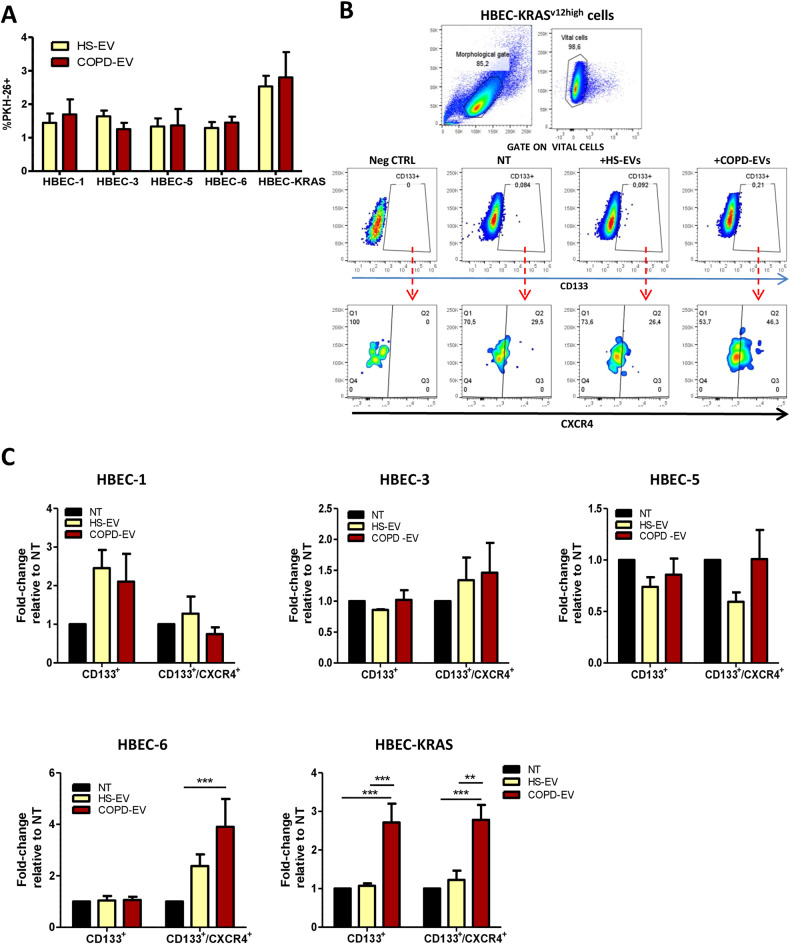
COPD-EVs increase the number of CD133^+^/CXCR4^+^ cells. **A** Flow cytometry analysis of the percentage of fluorescent HBEC-1, HBEC-3, HBEC-5, HBEC-6 and HBEC-KRAS^V12high^ cells after treatment with PKH26-labeled COPD- and HS-EVs (1 µg) (*n* = 5 per group). **B** Gating strategy used for the identification of CD133^+^ CICs and CD133^+^CXCR4^+^ MICs in HBEC cells after treatment with HS-EVs and COPD-EVs evaluated via flow cytometry. **C** Bar charts show the relative amount of CD133^+^ CICs and CD133^+^CXCR4^+^ MICs in HBEC-1 (*n* = 5), HBEC-3 (*n* = 3), HBEC-5 (*n* = 5), HBEC-6 (*n* = 5) and HBEC-KRAS^V12high^ after EVs treatment (*n* = 7).

To investigate the role of plasma EVs isolated from COPD subjects in fostering tumorigenic progression, we first evaluated the uptake of PKH26-labeled EVs on human immortalized bronchial epithelial cells HBEC-1, and differently transformed epithelial cell lines as HBEC-3, HBEC-5, HBEC-6, and HBEC-KRAS^V12high^ that model sequential steps of the neoplastic transformation process. In flow cytometry analysis, we observed a similar uptake of HS-EVs and COPD-EVs by all cell lines (Fig. 2A).

We next explored how COPD-EVs promote the acquisition of tumorigenic potential through the modulation of CIC phenotype in immortalized and transformed epithelial cells. Flow cytometry analysis proved a differential modulation of CD133^+^ cell subsets induced by COPD-EVs according to the specific genetic background of treated cells (Fig. 2B, C). In particular, COPD-EVs compared to HS-EVs significantly increased the subset of CD133^+^ CICs and specifically the subset of metastatic CD133^+^CXCR4^+^ cells only in HBEC-6 cells, harboring p53 and KRAS^V12^ mutation, and in HBEC-KRAS^V12high^ cells, whereas modest effect on CICs was observed in HBEC 1 immortalized cells and in transformed HBEC lines harboring sh-p53 or KRAS^V12^ oncogenic mutation only (HBEC 3 and 5) (Fig. 2C). These data suggest that COPD-EVs are more prone to foster the expansion of aggressive metastasis initiating cells in premalignant cells accumulating multiple oncogenic hits.

### HBEC-KRAS^V12high^ acquire tumorigenic properties following COPD-EVs treatment

**Fig. 3:**
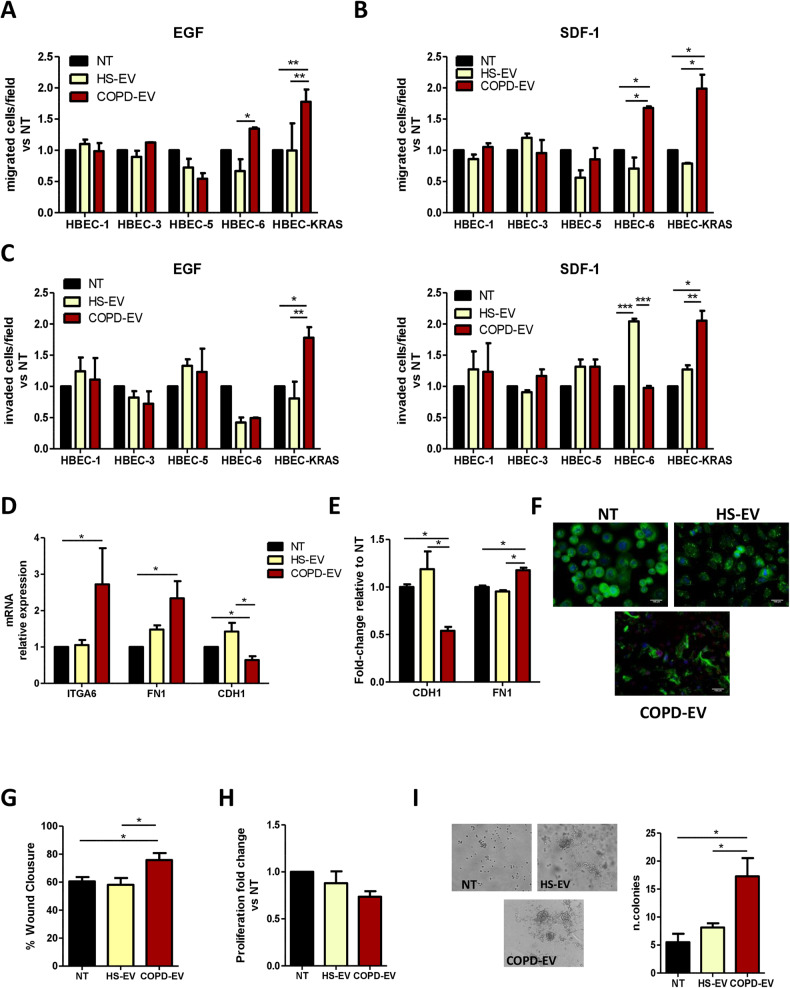
COPD-EVs induce the acquisition of an aggressive phenotype. **A** The migratory ability of HBEC cells after the indicated treatments with EVs towards an EGF gradient was evaluated in Transwell assay (*n* = 4). Migration data are expressed as the number of migrated cells compared to the number of migrated control cells. **B** Evaluation of the migration towards SDF-1 factor in HBEC cells after the treatment with HS-EVs or COPD-EVs. **C** The acquisition of invasive properties after EVs treatment was analyzed in HBEC cells following the gradient constituted by using EGF or SDF-1. Invasion data are expressed as the number of invaded cells versus the number of invaded control cells. **D** Expression level of genes involved in stemness maintenance and in EMT process (*n* = 5). B2m was used as housekeeping control. **E** Flow cytometry confirmed the induction of EMT in HBEC-KRAS^V12high^ after COPD-EVs considering E-cadherin (CDH1) and fibronectin (FN1) expression (*n* = 3). **F** Representative images of E-cadherin (green) and Snail2 (red) immunofluorescent staining of HBEC-KRAS^V12high^ after EV administration. **G** HBEC-KRAS^V12high^ migration ability after EVs administration (wound healing assay, *n* = 4). The Bar chart shows the percentage of wound compared to controls. **H** Analysis of 2D proliferation of HBEC-KRAS^V12high^ cells treated with EVs (*n* = 4). **I** 3D proliferation assay using VitroGel. Images show the colony formation ability of HBEC-KRAS^V12high^ cells treated with 15 µg of COPD- or HS-EVs. In all experiments, untreated cells (NT) were used as control. Data are expressed as mean and SEM.

To further characterize the acquisition of a more aggressive phenotype induced by COPD-EVs, we analyzed the migratory and invasive abilities of EV-treated HBEC subtypes using a transwell assay. After 24 h, the treatment with both HS-EVs and COPD-EVs did not affect the migration of HBEC-1, HBEC-3, and HBEC-5 neither towards EGF, chemoattractant for epithelial cells (Fig. 3A and Supplementary Fig. S1A), nor towards SDF-1, the specific chemoattractant for CXCR4^+^ cells (Fig. 3B and Supplementary Fig. S1B). Instead, the treatment with COPD-EVs significantly increased the migration of both HBEC-6 and HBEC-KRAS^V12high^ compared to controls in the two experimental conditions. Invasion assay revealed an increased invasive ability of COPD-EVs treated HBEC-KRAS^V12high^ (Fig. 3C and Supplementary Fig. S1C, D). Along with the acquisition of CICs phenotype and migrating/invasive properties, COPD-EVs treatment of HBEC-KRAS^V12high^ significantly up-regulated the expression of some stemness and mesenchymal genes, as Integrin-α6, fibronectin and SNAI2, with a concomitant down-modulation of E-cadherin (CHD1) compared to controls, indicating a possible induction of EMT (Fig. 3D and Supplementary Fig. S2A). We confirmed a similar trend in the modulation of these EMT markers induced by COPD-EVs also at protein levels, using flow cytometry (Fig. 3E and Supplementary Fig. S2B) and immunofluorescence (Fig. 3F). Conversely, no modulation in these genes was observed in the other tested cell lines (Supplementary Fig. S2C).

Based on these preliminary results, we focused our attention on HBEC-KRAS^V12high^ in which a more striking effect in the acquisition of a malignant phenotype was observed after COPD-EVs treatment. We further observed that the treatment with COPD-EVs significantly increased the percentage of wound closure compared to HS-EVs (% wound closure: COPD-EVs= 75.8%, SEM = 5.1%; HS-EVs= 58.2%, SEM = 4.8%; NT = 60.6%, SEM = 3.1%. *p* = 0.0451 vs. HS-EVs and *p* = 0.042 vs. NT) (Fig. 3G and Supplementary Fig. S3). Instead, proliferation assay showed that COPD-EVs did not affect the growth rate of HBEC-KRAS^V12high^, cultured as monolayer (Fig. 3H); however, COPD-EVs but not HS-EVs fostered the 3D-colony forming ability of HBEC-KRAS^V12high^, providing and indirect indication on the pro-tumorigenic features of COPD-EVs treated cells (Fig. 3I). Overall, these results suggest that COPD-EVs promote the increase of CD133^+^ cancer-initiating cells, in particular through the expansion of CD133^+^CXCR4^+^ MICs subset, along with the acquisition of pro-tumorigenic and invasive properties in HBEC-KRAS^V12high^ cells.

### HIF-1α is transferred from COPD-EVs to HBEC-KRAS^V12high^

**Fig. 4:**
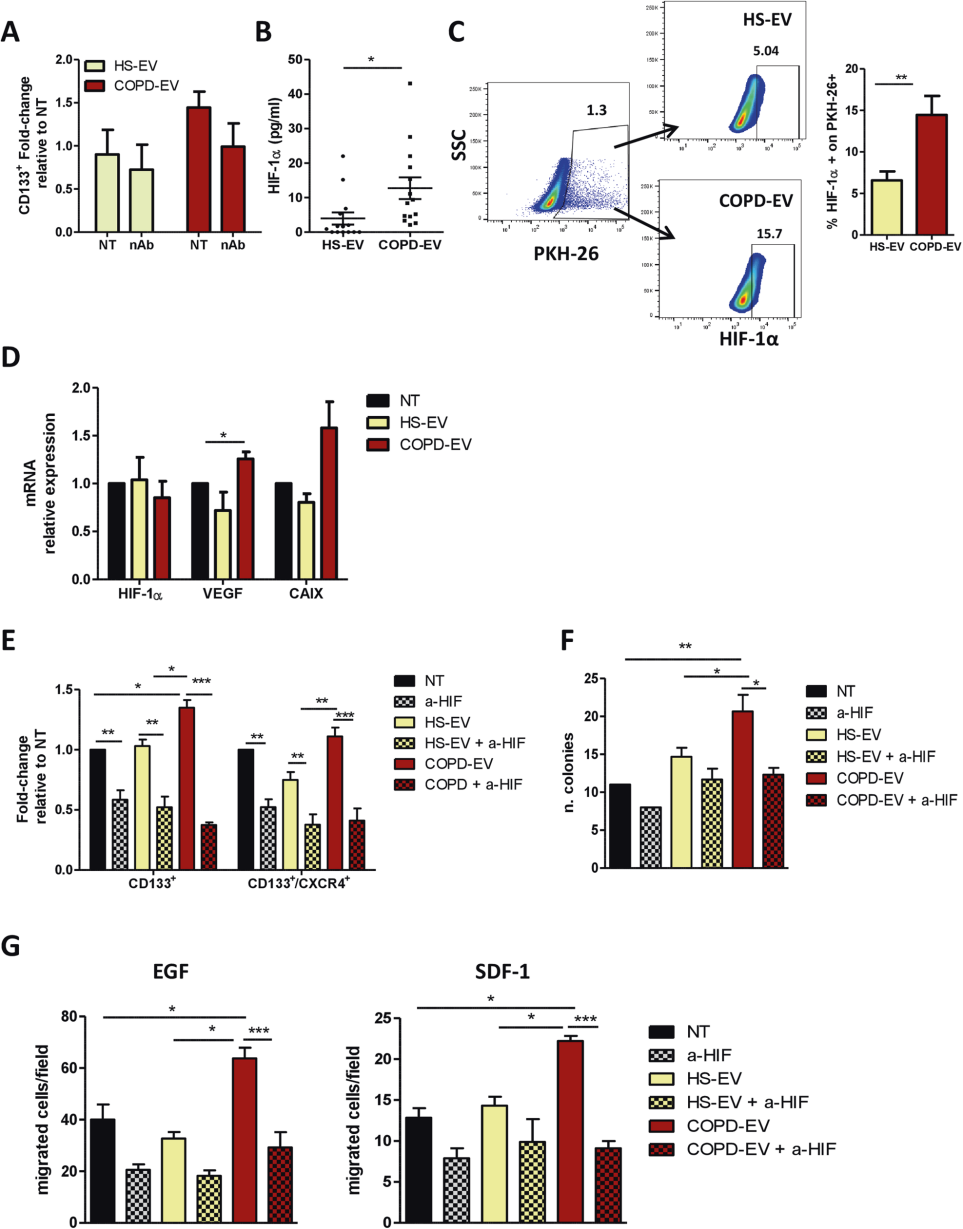
HIF-1α is transferred by COPD-EVs in recipient cells. **A** Analysis of CD133 modulation in HBEC-KRAS^V12high^ treated with EVs in the presence of SDF-1 neutralizing antibody (*n* = 3). **B** Quantification (pg/µl) of HIF-1α carried by COPD or HS-EVs via ELISA (*n* = 10). **C** On the left, representative dot plots show the higher positivity for HIF-1α in HBEC-KRAS^V12high^ treated with COPD-EVs compared to HS-EVs. On the right, the bar chart shows the percentage of HIF-1α in PKH26-labeled EV-treated HBEC-KRAS^V12high^ using flow cytometry (*n* = 7). **D** mRNA expression level of HIF-1α, VEGF and CAIX in EV-treated cells (*n* = 4). **E** Flow cytometry analysis of CD133^+^ and CD133^+^CXCR4^+^ cells after concomitant HIF-1α inhibition and EVs administration in HBEC-KRAS^V12high^ (*n* = 5). **F** Quantification of colonies constituted by HBEC-KRAS^V12high^ cells treated with 15 µg of COPD- or HS-EVs and concomitant HIF-1α inhibition in 3D culture condition. Untreated cells (NT) were used as a negative control. **G** Evaluation of migratory capabilities of HBEC-KRAS^V12high^ towards EGF and SDF-1 gradients after the treatment with HS- and COPD-EVs in the presence or not of HIF-1α inhibitor (*n* = 3). Data are expressed as mean and SEM.

To investigate the factors causing the increase of MICs subset and the acquisition of pro-tumorigenic properties induced by COPD-EVs we focused on SDF-1, the cytokine that activates CXCR4 downstream signaling pathways, also known to regulate CICs phenotype [30]. Treatment of HBEC-KRAS^V12high^ cells with neutralizing antibody against SDF-1 did not abrogate the modulation of CICs cells induced by COPD-EVs, indicating that SDF-1-CXCR4 activation axis was not central in COPD-EVs induced expansion of CICs (Fig. 4A). Since it has been demonstrated that COPD causes a hypoxic lung microenvironment, we decided to analyze inside EVs the amount of the transcription factor HIF-1α, the master regulator of the cellular response to hypoxia. HIF-1α level, evaluated via ELISA, was significantly lower in HS-EVs (2.1 pg/ml, SEM = 1.5 pg/ml) than in COPD-EVs (7.5 pg/ml, SEM = 2.0 pg/ml) (*p = 0.03) (Fig. 4B). The transfer of HIF-1α into recipient cells via EVs was evaluated by treating HBEC-KRAS^V12high^ with PKH-labeled-EVs. Intracellular level of HIF-1α in PKH-fluorescent positive cells treated with COPD-EVs was 2.2-fold increased compared to the HS-EV treatment (*p* < 0.01) (Fig. 4C). To further elucidate whether the increase of HIF-1α in recipient cells was actually due to EVs protein shuttling or to the induction of endogenous transcript, levels of HIF-1α mRNA were evaluated after EVs treatment of HBEC-KRAS^V12high^. No differences in HIF-1α transcription were observed between the two groups (Fig. 4D), indicating that HIF-1α was not induced into recipient cells but shuttled by EVs. Moreover, the transfer of HIF-1α from COPD-EVs induced the transcription of known downstream targets such as VEGF and CAIX in recipient cells (Fig. 4D). To assess the role of HIF-1α in the induction of MICs phenotype caused by COPD-EVs, we exploited a selective HIF-1α inhibitor, PX-478, before EVs treatment. HIF-1α inhibition completely abrogates the expansion of HBEC-KRAS^V12high^ CD133^+^ CICs induced by COPD-EVs, especially acting on CD133^+^CXCR4^+^ MICs (Fig. 4E). Coherently with effects on tumorigenic CICs subset, the blockade of HIF-1α transfer after COPD-EVs treatment reduced the number of 3D-colonies (Fig. 4F). Furthermore, the inhibition of HIF-1α reversed the pro-migratory effect, towards both SDF-1 and EGF chemoattractants, induced by COPD-EVs (Fig. 4G).

### HIF-1α shuttle induces CXCR4 modulation and CD133^+^ stem cells

**Fig. 5:**
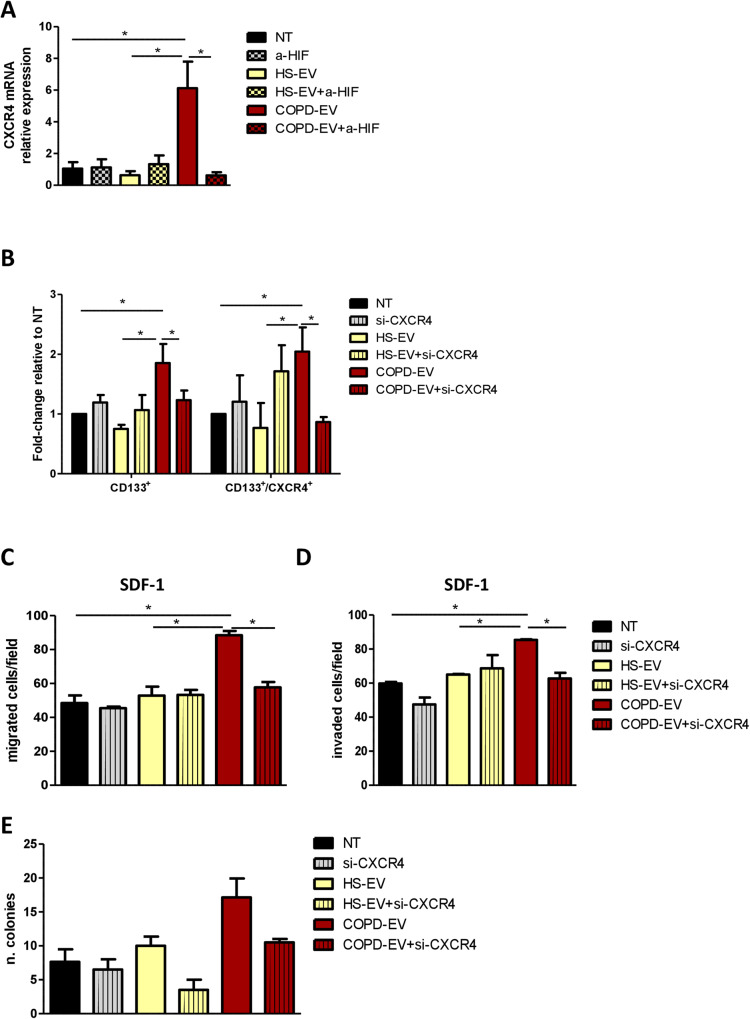
HIF-1α shuttle induces CXCR4 modulation and CD133^+^ stem cells. **A** The increase of mRNA of CXCR4 in HBEC-KRAS^V12high^ induced by COPD-EVs was abrogated by the use of HIF-1α inhibitor. **B** The silencing of CXCR4 reversed the modulation of CD133^+^ CICs and CD133^+^CXCR4^+^ MICs in COPD-EVs treated. In all experiments, untreated cells were used as control. **C** The silencing of CXCR4 in HBEC-KRAS^V12high^ cells reversed the migratory capability gained by cells treated with COPD-EVs. **D** Pro-invasive properties given by the treatment with COPD-EVs to HBEC-KRAS^V12high^ cells were abolished by the silencing of CXCR4. **E** Colonies formation induced by COPD-EVs treatment in HBEC-KRAS^V12high^ cells was inhibited by CXCR4 silencing. *p < 0.05. Data are expressed as mean and SEM.

Since HIF-1α controls directly CXCR4 expression that in turn modulates CICs phenotype, we investigated its possible involvement as mediator of COPD-EVs induced-CICs effect. We confirmed that CXCR4 transcription was significantly increased in HBEC-KRAS^V12high^ cells after COPD-EVs treatment and that the inhibition of HIF-1α completely abrogated this effect (Fig. 5A). Notably, no CXCR4 modulation was observed after HS-EVs administration. Furthermore, CXCR4 silencing in HBEC-KRAS^V12high^ recipient cells completely prevented the increase of CD133^+^ and CD133^+^CXCR4^+^ subsets caused by COPD-EVs (Fig. 5B). Of note, the blockade of CXCR4 in HBEC-KRAS^V12high^ reverted the capacity of COPD-EVs to promote migration (Fig. 5C) and invasion (Fig. 5D) of these cells. Furthermore, the number of 3D colonies induced by COPD-EVs was reduced after CXCR4 silencing in recipient cells (Fig. 5E).

All together these data support the crucial role of CXCR4 as a mediator of hypoxia-induced modulation of MIC phenotype.

### Hypoxia plays a key role in MIC induction and in cancer development

**Fig. 6:**
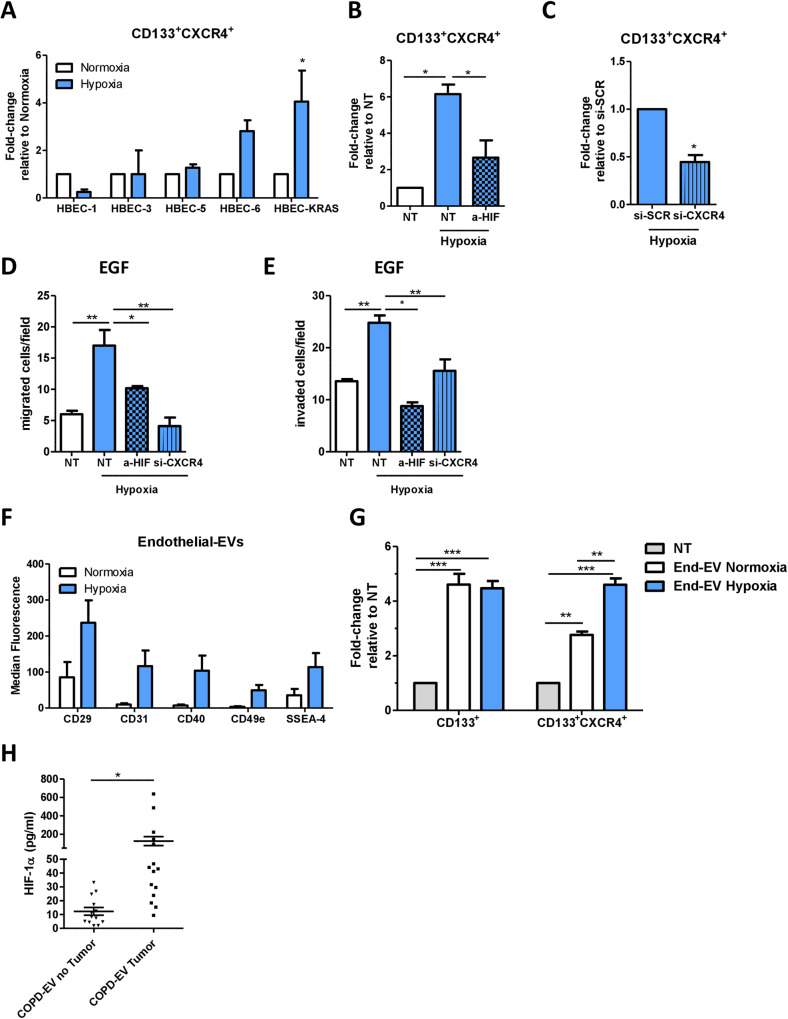
Hypoxia induces MIC expansion. **A** Relative amount of CD133^+^CXCR4^+^ MICs in HBEC-1, HBEC-3, HBEC-5, HBEC-6, and HBEC-KRAS^V12high^ in normoxic or hypoxic condition (*n* = 3). **B** In hypoxic condition, the treatment with anti-HIF-1α of HBEC-KRAS^V12high^ inhibits the expansion of CD133^+^CXCR4^+^ MICs compared to untreated cells cultured in hypoxia (*n* = 4). **C)** MICs expansion in HBEC-KRAS^V12high^ was abrogated by inducing the silencing of CXCR4 (*n* = 4). **D** Migration assay towards an EGF gradient using HBEC-KRAS^V12high^ pre-treated with HIF-1α inhibitor or CXCR4 siRNA and cultured 48 h in hypoxic condition prior to perform the assay. **E** Invasion assay towards an EGF gradient using HBEC-KRAS^V12high^ pre-treated with HIF-1α inhibitor or CXCR4 siRNA and cultured 48 h in hypoxic condition prior to seed cells for the assay. **F** Evaluation of endothelial markers by using MACSPlex kit on EVs released by endothelial cells cultured in both normoxic and hypoxic conditions (*n* = 3). **G** Flow cytometry analysis on HBEC-KRAS^V12high^ to evaluate the possible expansion of CIC and MIC subsets following the treatment with END-EVs Normoxia and END-EVs Hypoxia. **H** ELISA performed to evaluate the level of HIF-1α within EVs isolated from COPD volunteers that subsequently developed (*n* = 15) or not (*n* = 13) lung cancer. *p < 0.05, **p < 0.01, ***p < 0.001. Data are expressed as mean and SEM.

To further demonstrate the central role of hypoxia in the regulation of MIC phenotype, HBEC-1, HBEC-3, HBEC-5, HBEC-6, and HBEC-KRAS^V12high^ were incubated for 72 h at 2% oxygen and the modulation of CD133^+^CXCR4^+^ was evaluated. Hypoxia induced a 4-fold increase of MIC cells only in HBEC-KRAS ^V12high^ cells (*p = <0.05) unlike other cell lines tested (Fig. 6A). Once again, the HIF-1α blocking (Fig. 6B) or the CXCR4 silencing (Fig. 6C) prevented the expansion of MICs in HBEC-KRAS^V12high^ in hypoxic conditions. Finally, we demonstrated that hypoxia promoted migratory and invasive capacities of HBEC-KRAS^V12high^ that are abrogated by the inhibition of HIF-1α or CXCR4 in recipient cells towards an EGF gradient (Fig. 6D, E and Supplementary Fig. S4). These results corroborate the observation that the cellular response to hypoxia is central in promoting MIC expansion but only in transformed epithelial cells accumulating multiple oncogenic hits, similar to what we previously observed after COPD-EVs treatment.

Our MACSPlex analysis in Fig. 1E indicated a significant enrichment of endothelial markers in circulating EVs obtained from COPD subjects, suggesting their endothelial cell origin. To assess whether hypoxic environment occurring in COPD subjects promotes EVs release by endothelial cells, we cultured endothelial cells in hypoxic and standard oxygen culture conditions and we next recovered and analyzed EVs via MACSPlex. Hypoxia induced an increase in endothelial markers on the surface of EVs compared to normal condition (Fig. 6F), confirming that hypoxia boosted the release of EVs from endothelial cells.

We verified that EVs released by endothelial cells, maintained both in normoxic (END-EVs Normoxia) and in hypoxic conditions (END-EVs Hypoxia), have a similar ability to increase the total number of CD133^+^ cells in HBEC-KRAS^V12high^ recipient cells, but EVs released by endothelial cells under hypoxia can specifically expand the pool of CD133^+^CXCR4^+^ cells, suggesting their unique ability to activate CXCR4 pathway (Fig. 6G).

Finally, to confirm the involvement of hypoxia in cancer development, we analyzed the level of HIF-1α inside EVs from COPD volunteers, who subsequently developed or not lung cancer. We found significant higher levels of HIF-1α (125.8 pg/ml, SEM = 48.66 pg/ml) in EVs from COPD subjects who subsequently developed cancer (i.e. before CT scan detection) than COPD subjects who remained cancer free for at least three years (12.34 pg/ml, SEM = 2.835 pg/ml) (*p = 0.04) (Fig. 6H).

Taken together, these results highlight the strong involvement of COPD-derived hypoxia in inducing the release of EVs by endothelial cells and the potential role of HIF-1α within EVs in fostering lung cancer development that may be exploited as biomarker of lung cancer risk.

## Discussion

Smoking leads to an increase of inflammation in the lungs that modifies the phenotype of epithelial and stromal cells [28]. COPD is associated with persistent respiratory symptoms and elevated inflammation levels [31]. Several evidences link the persistence of COPD with a higher incidence of lung cancer [32]. However, only 1 % of COPD subjects develop lung cancer indicating the existence of a complex mechanism behind the increased risk of lung carcinogenesis [33].

Recently, EVs emerged as important regulators of COPD and lung cancer [34]. It has been reported that COPD and inflammation modifies the phenotype of circulating EVs either considering surface markers or cargo [23]. Indeed, this pathological condition stimulates the release of EVs from endothelial cells and monocytes in the blood [35], whereas in the broncho-alveolar lavage vesicles expressing endothelial and macrophages markers were detected in COPD smokers compared to non smoker subjects [36]. In both of these studies the amount of vesicles correlates with the severity of COPD. Here, we confirmed a higher presence of endothelial-derived EVs in COPD subjects but we also found a general increase of surface markers derived from immune cells suggesting a possible involvement of adaptive immune response in the pathogenesis of the disease.

Hypoxia-inducible factors (HIFs), strongly deregulated in COPD disease, were also deregulated in several solid tumors and modulated different signaling pathways such as proliferation, EMT, metastatization and also resistance to therapy [37]. It has been demonstrated in experimental mouse models that COPD-like airway inflammation can lead to lung cancer promotion in a context of KRAS mutant epithelial cells [38, 39]. Interestingly, we proved that both in vitro hypoxic conditions and HIF-1α shuttled from COPD-EVs to recipient cells induce aggressive cancer phenotype and malignant progression through the increase of MICs population, but only in the context of an already activated genetic background of epithelial cells carrying different oncogenic hits, including amplification of KRAS^V12^.

HIF-1α and hypoxia are involved in EV biogenesis both in normal and pathological conditions and are also implicated in the regulation of EV cargo such as protein and miRNAs [40]. It has been shown that nasopharyngeal carcinoma-derived vesicles induced EMT phenotype in recipient cell through the direct transfer of HIF-1α and this modulation increase the migratory and pro-metastatic ability of these cells [41]. Moreover, HIF-1α directly regulates the transcription of CXCR4, a chemokine receptor involved in cancer cell migration, invasion and in the maintenance of CICs in different solid tumors [42, 43]. In NSCLC, we found that CXCR4 pathway is activated in the specific subset of CD133^+^ CICs endowed with the highest metastasis-initiating ability [13]. We also previously showed that microenvironmental stimuli modulate lung MICs, through the induction of the EMT process [13]. Here, we demonstrated that the shuttle of HIF-1α contained in EVs from cancer-free individuals activates the transcription of CXCR4 that in turn increases the frequency of mesenchymal and disseminating CD133^+^CXCR4^+^ MICs. This increase was linked to the acquisition of tumorigenic, disseminating and mesenchymal properties by already transformed epithelial cells as functionally evaluated through in vitro assays.

We have already demonstrated that the transfer of plasma EV’s cargo from lung cancer subjects [44] or from individuals with a high risk of lung cancer [45] modulated the phenotype of premalignant epithelial cells through shuttling of c-Myc. Here, we show that COPD alters the protein content of EVs and that HIF-1α/CXCR4 axis activation caused by COPD-EVs in non-tumorigenic epithelial cells leads to the acquisition of malignant features and cancer progression.

Of clinical relevance, we observed that levels of HIF-1α in EVs from COPD subjects who subsequently developed cancer are significantly higher than in COPD subjects who remained cancer-free. These data suggest that in the context of COPD, analysis of HIF-1α in EVs cargo could be exploited to provide a novel biomarker to identify individuals at the highest risk of developing lung cancer.

## Materials and methods

### Cell lines

Immortalized human bronchial epithelial cells (HBEC-1: hTERT+Cdk4; HBEC-3: hTERT+Cdk4 + KRAS^V12^; HBEC-5: hTERT+Cdk4+sh-p53; HBEC-6: hTERT+Cdk4+sh-p53 + KRAS^V12^; HBEC-KRAS^V12high^: hTERT+Cdk4+sh-p53+KRAS^V12high^) were provided by Prof. J.D. Minna (UT Southwestern, TX) [46]. Cells were cultured in Keratinocyte–Serum-Free Medium supplemented with 5 ng/ml human recombinant EGF and 50 μg/ml bovine pituitary extract (K-SFM; Thermo Fisher Scientific, Waltham Massachusetts, USA).

More details on cells treatments are provided in Supplementary Methods.

### Clinical specimens

Plasma samples were collected from heavy smoker volunteers (age 50–75 years) enrolled in a low dose CT screening trial performed at our institution (BioMild Trial, ClinicalTrials.gov: NCT02247453) [47]. Clinical characteristics of heavy smokers volunteers cancer free and heavy smokers volunteers who developed lung cancer are summarized in Table 1. Plasma collection was approved by Internal Review and Ethics Boards of Istituto Nazionale Tumori of Milan (INT 11-21).

**Table 1:** Clinical characteristics of heavy-smokers volunteers.

	HS-EV (*n* = 10)	COPD-EV (*n* = 23)	COPD-EV Tumor (*n* = 15)
*Gender*			
Male	7 (70%)	14 (61%)	10 (67%)
Female	3 (30%)	9 (39%)	5 (33%)
Age (years)			
	57.8 ± 17,9	67 ± 5.4	71,1 ± 6.6
*Smoking status*			
Current	9 (90%)	18 (78%)	10 (67%)
Former	1 (10%)	5 (22%)	5 (33%)
Smoking habit (Pack-Year index)			
	39.3 ± 10.6	49.1 ± 17.5	51 ± 13.1
*Emphysema*			
Yes	1 (10%)	13 (57%)	5 (33%)
No	9 (90%)	10 (43%)	10 (67%)
Forced Expiratory Volume 1 (FEV1%)			
	90.9 ± 26,4	83.1 ± 16	89.1 ± 15,1
*COPD grade*			
0	10 (100%)	–	–
I	–	15 (65%)	11 (73%)
II	–	7 (30%)	3 (20%)
III	–	1 (5%)	1 (7%)
*Tumor subtypes*			
ADC	–	–	9 (60%)
SCC	–	–	2 (13%)
Other	–	–	4 (27%)
*Stage*			
I-II			10 (67%)
III-IV			5 (33%)

*HS* heavy smokers volunteers, *COPD* heavy smokers volunteers with COPD, *COPD-Tumor* heavy smokers volunteers with COPD who developed tumor during the screening trial.

### EVs isolation and characterization

EVs were isolated from the plasma of heavy smokers and COPD volunteers by ultracentrifugation at 120,000 × *g* at 4 °C for 90 min using a TLA-100.3 fixed-angle rotor in a TL-100 ultracentrifuge (Beckman Coulter, Brea, CA, USA). EV-enriched pellet was washed in filtered phosphate-buffered saline (PBS; Thermo Fisher Scientific) centrifuged at 120,000 × *g* for 60 min at 4 °C to eliminate impurities and later resuspended in PBS. The protein content of purified EVs was verified by Bradford assay.

For EVs isolation from conditioned media (CM), HUVEC were maintained in serum-free medium for 72 h in normal oxygen condition or in 2 % oxygen condition at 37 °C. Then, the CM was collected and centrifuged at 3200 × *g* for 25 min in order to remove cell debris. EVs isolation was performed following the same protocol used for plasma.

EVs concentration and size distribution were evaluated by using a NanoSight NS300 instrument (Malvern Panalytical) whereas surface markers profile of plasma EVs was characterized by MACSPlex Kit (Milteny Biotec), as detailed in Supplementary Methods.

### Flow cytometry

#### EVs analysis

EVs (15 µg) were incubated with 10 µl of Aldehyde/Sulfate latex beads, 4% w/v 4 µm (Invitrogen, Thermo Fisher Scientific) for 15 min at room temperature (RT) and later incubated overnight at 4 °C in PBS. After incubation, 100 mM glycine was added for 30 min at RT and then samples were stained with antibodies, as detailed in Supplementary Methods.

#### HBEC analysis

Quantification of CICs in different cell lines was performed 72 h post-treatment with EVs. Cells were resuspended in staining buffer (PBS 1X + 0.5 % BSA) and incubated with PE-conjugate anti-human CD133 antibody (Miltenyi Biotec) and APC-conjugate anti-human CXCR4 (BD Pharmingen) antibody for 20 min at 4 °C. After washing, cells were incubated with 7-AAD live/dead solution (Thermo Fisher Scientific) and the acquisition was performed using a FACSCanto II flow cytometer (BD Pharmingen).

### Wound-healing assay

Wound-healing assay was performed to evaluate the migratory ability of HBEC-KRAS^V12high^. 5×10^4^ HBEC-KRAS^V12high^, pre-treated with 15 µg of HS- or COPD-EVs, were seeded on both sides of Culture Insert 2-well in µ-Dish 35 mm (Ibidi). After 24 h, the culture insert was removed and 1 ml of Keratynocyte-SFM was added in each well. The cell-free gap was monitored and photographed immediately and after 3 h using a JuLI Stage Real-Time Cell History Recorder (NanoEntek). The percentage of wound closure was evaluated in 5 random fields using the ImageJ Software.

### 3D Proliferation assay

The 3D proliferation assay was performed to evaluate the ability of HBEC-KRAS^V12high^ to grow as colonies. The assay was performed using Vitrogel 3D (TheWell Bioscience, USA). A 1:1 VitroGel mix was added to 24-well plates and incubated for 20 min at 37 °C. Then, 5 × 10^4^ of pretreated HBEC-KRAS^V12high^ were seeded on the VitroGel’s layer. After 14 days, the number of colonies was counted in 5 random fields per condition.

### Statistical analysis

The statistical analysis was performed using the Graphpad Prism 5 software. Statistically significant differences were determined with Student’s t‐test when comparing two groups or the ANOVA test for multiple comparisons.

### Supplementary information

Supplementary materials

Reproducibility Checklist

## Data Availability

All datasets generated and analyzed during this study are included in this published article and its Supplementary Information files. Additional data are available from the corresponding author on reasonable request.
