# Supplementary material for: Extracellular vesicles from subjects with COPD modulate cancer initiating cells phenotype through HIF-1α shuttling
Source: Cell Death Dis. 2023 Oct 14;14(10):681. doi: 10.1038/s41419-023-06212-1 (PMC10576796; doi:10.1038/s41419-023-06212-1)
Supplement: Supplementary file 1 — Supplementary materials [file 41419_2023_6212_MOESM1_ESM.pdf]

**Cell Treatments**

To evaluate the role of HIF-1α, HBEC-KRAS$^{V12high}$ cells were treated with 15 µg of EVs from heavy-smoker individuals (HS-EVs) and from COPD patients (COPD-EVs) with the concomitant addiction of PX-478 2HCl, a selective HIF-1α inhibitor (10 µM, Selleckchem). In experiments where the SDF-1/CXCR4 signaling pathway was investigated, neutralizing antibody anti-SDF-1 (MAB-310, R&D system) was used at 5 µg/ml, simultaneously with EV administration and CD133$^{+}$ modulation was evaluated after 72 h.

Primary human umbilical vein endothelial cells (HUVECs) were purchased from Lonza (Lonza, Basel, Switzerland) and cultured in Endothelial Cell Growth Basal Medium-2 (EBM-2; Lonza, Basel, Switzerland).

For hypoxia experiments, cells were maintained in a Whitley H25 Hypoxystation (Don Whitley Scientific Limited, UK) with 2% oxygen at 37 °C for 48 h.

In experiments where HBEC-KRAS$^{V12high}$ were treated with EVs obtained from the conditioned medium of HUVEC cells kept in normoxic or hypoxic condition, 10 µg of EVs were used.

***EVs characterization***

NanoSight: The EVs concentration and the size distribution were evaluated by using a NanoSight NS300 instrument (Malvern Panalytical). Videos obtained were analyzed using NTA 3.2 software. The morphology of EVs was determined using a Zeiss LIBRA 200FE transmission electron microscope with an in-column second-generation Omega filter. The size of EVs was quantified by analyzing 100 EVs using the iTEM imaging platform.

MACSPlex analysis: To determine the surface marker profile of plasma EVs, MACSPlex Kit (Milteny Biotec, Bergisch-Gladbach, Germany) was used following manufacturer's instructions. Briefly, 15 µg of EVs were incubated with MACSPlex Exosome Capture Beads which are fluorescently labeled beads coated with 37 different antibodies that recognize corresponding surface epitopes on EVs. Epitopes recognized by beads are listed below.

| **Epitope** | **Isotype** |
| --- | --- |
| CD3 | mouse IgG2a |
| CD4 | mouse IgG2a |

| | |
|---|---|
| CD19 | mouse IgG1 |
| CD8 | mouse IgG2a |
| HLA- DRDPDQ | recombinant human IgG1 |
| CD56 | recombinant human IgG1 |
| CD105 | recombinant human IgG1 |
| CD2 | mouse IgG2b |
| CD1c | mouse IgG2a |
| CD25 | mouse IgG1 |
| CD49e | recombinant human IgG1 |
| ROR1 | mouse IgG1κ |
| CD209 | mouse IgG1 |
| CD9 | mouse IgG1 |
| SSEA-4 | recombinant human IgG1 |
| HLA-ABC | recombinant human IgG1 |
| CD63 | mouse IgG1κ |
| CD40 | mouse IgG1κ |
| CD62P | recombinant human IgG1 |
| CD11c | mouse IgG2b |
| CD81 | recombinant human IgG1 |
| MCSP | mouse IgG1 |
| CD146 | mouse IgG1 |
| CD41b | recombinant human IgG1 |
| CD42a | recombinant human IgG1 |
| CD24 | mouse IgG1 |
| CD86 | mouse IgG1 |
| CD44 | mouse IgG1 |
| CD326 | mouse IgG1 |
| CD133/1 | mouse IgG1κ |

| | |
|---|---|
| CD29 | mouse IgG1κ |
| CD69 | mouse IgG1κ |
| CD142 | mouse IgG1κ |
| CD45 | mouse IgG2a |
| CD31 | mouse IgG1 |
| CD20 | mouse IgG1 |
| CD14 | mouse IgG2a |

Subsequently, complexes EVs-Capture beads are incubated with MACSPlex Exosome Detection Reagent cocktail, bearing on their surface antibodies against CD9, CD63 and CD81. Samples were incubated at RT in the dark for 1 h, washed twice with 500 µl of MACSPlex Buffer and acquired by flow cytometry (FACSCanto II flow cytometer) and analyzed with FlowJo software.

**List of Antibodies**

| **Antibody** | **Clone** | **Fluorescence** | **Company** |
|---|---|---|---|
| Anti-human CD9 | HI9a | FITC | Biolegend, #312104 |
| Anti-human CD63 | H5C6 | APC | Biolegend, #353008 |
| Anti-human CD81 | TAPA-1 | PE-Cyanine7 | Biolegend, #349512 |
| Anti-human CD133 | AC133 | PE | Miltenyi Biotech |
| Anti-human CXCR4 | 12G5 | APC | BD Pharmingen |
| Anti-human HIF-1α | 241812 | AF 405 | R&D SYSTEMS |
| Anti-human E-cadherin | 67A4 | Alexa Fluor 488 | BioLegend #324110 |
| Anti-human N-cadherin | Polyclonal IgG | APC | R&D SYSTEMS #FAB6426A |
| Anti-mouse/human Integrin α6 | EBioGoH3 | Super Bright 436 | Invitrogen #2081737 |
| Anti-human Fibronectin | 10/Fibronectin | AF 647 | BD Pharmingen #563098 |
| Anti-human Vimentin | VI-RE/1 | PE | Abcam #ab49918 |

**Flow Cytometry for HIF-1α analysis**

To evaluate the transfer of HIF-1α carried by EVs into recipient cells, HBEC-KRAS$^{V12high}$ cells were treated for 24 h with 15 µg of PKH26-labelled–EVs. Then, cells were collected and fixed with the Fixation Buffer (BD Bioscience) for 30 min at 4 °C. After the fixation, cells were permeabilized with the Permeabilization buffer (eBioscience) and stained with AF 405–conjugate anti-human HIF-1α antibody (clone: 241812 cat. n: #IC1935V-100UG, R&D SYSTEMS) for 30 min at 4 °C. After the staining, cells were washed with TF PERM/WASH buffer 5X (eBioscience) and the acquisition was performed using a FACSCanto II flow cytometer. All flow cytometry analyses were performed by FlowJo software.

**Flow Cytometry for EMT marker**

To assess if COPD- and HS-EVs treatment could affect the expression of markers involved in the EMT process in HBEC-KRAS$^{V12high}$ cells, the expression of CDH1, CHD2, ITGA6, FN1 and VIM were analyzed by flow cytometry. For CDH1, CHD2, ITGA6 staining, cells were collected and stained with antibodies of interest, reported in the List of antibodies, for 20 min at 4 °C. After washing, cells were incubated with 7-AAD live/dead solution (Thermo Fisher Scientific) prior to the acquisition. For the intracellular staining of FN1 and VIM, the protocol reported for HIF-1α flow cytometry experiments was performed as reported above, but using antibodies of interest, reported in the List of antibodies. Samples were acquired using a FACSCanto II flow cytometer. All flow cytometry analyses were performed by FlowJo software.

***Western Blot***

EVs (30 µg) were incubated with RIPA buffer and protease inhibitors cocktail for 30 min on ice. Protein samples were then run on a 4-12 % polyacrylamide gel (Thermo Fisher Scientific) and transferred to a nitrocellulose transfer membrane using the iBlot 2 Gel Transfer Device (Thermo Fisher Scientific). Aspecific binding sites were blocked by incubating membranes with 5 % non-fat milk in T-TBS 1X. After the blocking, membranes were incubated overnight with primary antibodies of interest. For this study, primary antibodies used with their dilution were: rabbit anti-human CD9 monoclonal antibody 1:1000 (cat. n: 74220, Cell Signaling Technology, Danvers, Massachusetts, USA), rabbit anti-human CD81 monoclonal

antibody 1:1000 (cat. n: 10037, Cell Signaling Technology), rabbit anti-human Tsg101 monoclonal antibody 1:1000 (cat. n: 72312, Cell Signaling Technology) and mouse anti-human ApoA1 monoclonal antibody 1:1000 (cat. n. number: 3350, Cell Signaling Technology). After 3 washing with T-TBS 1X, membranes were incubated with HRP-conjugated goat anti-rabbit IgG antibody 1:5000 (cat n: 7074, Cell Signaling Technology) or HRP-conjugated goat anti-mouse antibody 1:5000 (cat n: 31340, GE Healthcare Life Sciences, USA). Signals were detected via an enhanced chemiluminescence reaction (GE Healthcare Life Sciences, USA) in a MINI HD9 Western Blot Imaging System (Cleaver Scientific, UK). Images of original western blots for CD9, CD81, TSG101 and ApoA1 are shown in Supplemental Fig. S2A-D

***Fluorescence membrane staining of EVs for uptake experiments***

HS-EVs and COPD-EVs (15 µg) were labeled with the red fluorescence dye PKH26 (Sigma-Aldrich), a lipophilic dye used for the general labeling of cell membrane. Briefly, EVs were incubated with 1 µl of PKH26 dye diluted in 1 ml PBS for 5 min at RT. The dye excess was removed by washing EVs by ultracentrifugation at 120 000 × g for 60 min at 4 °C. Finally, labeled EVs were resuspended in filtered PBS and stored at -80 °C as 1 µg/aliquots.

***Migration and Invasion Assay***

For the migration assay, $5x10^4$ cells were plated into a 6 well plate and treated with 15 µg of HS-EVs or COPD-EVs. After 24 h, $1x10^5$ of treated cells were resuspended in 100 µl of RPMI free medium and then seeded into the top chamber of FluoroBlok 24 well cell culture inserts with 8 µm pore size (Corning, Glandale, AZ, USA). Instead, in the lower chamber, 750 µl of Keratynocite-SFM with EGF (Epithelial Grow Factor 100 ng/ml) or SDF-1 (50 ng/ml) was added. After 24 h, migrated cells were fixed with methanol 30% and their nuclei stained with VECTASHIELD Antifade Mounting Medium with DAPI (Vector Laboratories, Newark, CA, USA). The numbers of migrated cells (3 random fields/condition) were counted through fluorescence microscopy. For the invasion assay, inserts were coated with Matrigel (Becton Dickinson, Franklin Lakes, NJ, USA) and the assay was stopped after 48 h.

***Quantitative Real-Time PCR***

cDNA was synthesized from 500 ng RNA extracted from cell pellets by using a Maxwell RCS Instrument for automated RNA extraction (Promega Corporation). RT-PCR was performed using Taqman Universal Master Mix II (Thermo Fisher Scientific). The relative quantification of expression levels of selected genes was performed using B2m as the endogenous gene control. The data were calculated as $2^{\wedge}$-(ddCT) methods.

***CXCR4 silencing transfection***

For in vitro transfection experiments, $5x10^4$ HBEC-KRAS$^{V12high}$ cells were seeded in 6 well plate and transfected with 50 nM esiRNA1 human CXCR4 (EHU022821) or esiRNA Universal Negative Control with Lipofectamine 2000 according to the manufacturer's instruction. After 24 hours, cells were harvested for flow cytometry experiments.

***ELISA***

The quantity of HIF-1α inside 45 µg of EVs from HS and COPD individuals was evaluated by using the HIF-1α Kit (Raybiotech) following the manufacturer's instruction. The absorbance at 450 nm was measured using the Infinite M1000 Tecan spectrophotometer microplate reader (Tecan Group Ltd).

***Immunoflorescence***

HBEC-KRAS$^{V12high}$ cells ($5x10^5$) were cultured for 24 h in 8-well chamber slides (cat.n.:154534PK; ThermoFischer ) and fixed with PFA 4% (cat.n.:FC004; R&D System) for 20 min at RT. Cells were then permeabilized with Triton X-100 0.3% for 10 min at RT (cat.n.:T8787; Thermo Scientific Chemicals) and blocked with a Bovine Serum Albumin 2% added with Normal Goat Serum 5% solution for 60 min at RT. After blocking, cells were incubated with mouse anti–human E-Cadherin IgG2α,κ antibody, dilution 1:50 (cat.n.:610181; BD Trasduction Laboratory) and rabbit anti–human SNAI2 monoclonal antibody, dilution 1:400 (cat.n.:C19G7; Cell Signaling,) for 1h at RT. Then, cells were incubated with the secondary antibody Alexa Fluor 488 goat anti–mouse IgG polyclonal antibody (cat.n.:A11001; ThermoFisher) or Alexa Fluor 555 goat anti–rabbit IgG polyclonal antibody (cat.n.:A21428, ThermoFisher), and incubated for 45 min at RT

protected from light. For nuclei staining, a DAPI 600 nM was used (ThermoFisher). Slides were finally visualized with the tissue imager Vectra Polaris Automated Quantitative Pathology Imaging System (PerkinElmer, Massachusetts, USA). Acquired Images were analized with ImageJ software.

**SUPPLEMENTAL FIGURE LEGENDS**

**Supplemental Figure 1: Representative images of migration and invasion assay using Hbec-1, -3, -5, -6 and -KRAS$^{V12high}$ cells after COPD- and HS-EVs treatment.** Migration assay were performed using both EGF **(A)** and SDF-1 **(B)** gradients. Also for invasion assay EGF **(C)** and SDF-1 **(D)** were used as chemoattractants.

**Supplemental Figure 2: Expression levels of genes involved in stemness and in EMT process**. **A)** mRNA relative expression of staminal and EMT genes evaluated in HBEC-KRAS$^{V12high}$ cells left untreated (NT) or treated with COPD- and HS-EVs. **B)** Flow cytometry analysis to evaluate protein levels of CHD2, VIM and ITGA6. **C)** Real-time PCR analysis on stemness and EMT genes performed on HBEC-1, -3, -5, -6 samples after the treatment with COPD-EVs or HS-EVs. Data are expressed as mean and SEM.

**Supplemental Figure 3: Representative images of wound healing assay.**

**Supplemental Figure 4: Representative images of migration and invasion assay performed in hypoxic condition. A)** Migratory capacities of HBEC-KRAS$^{V12high}$ cells were assessed after the inhibition of HIF-1α or CXCR4 towards an EGF gradient. **B)** Invasion assay was performed using HBEC-KRAS$^{V12high}$ cells treated with HIF-1α or CXCR4 inhibitor. Untreated cells (NT) in normoxic and hypoxic conditions were used as controls in both assays.

## Supplemental Figure 1

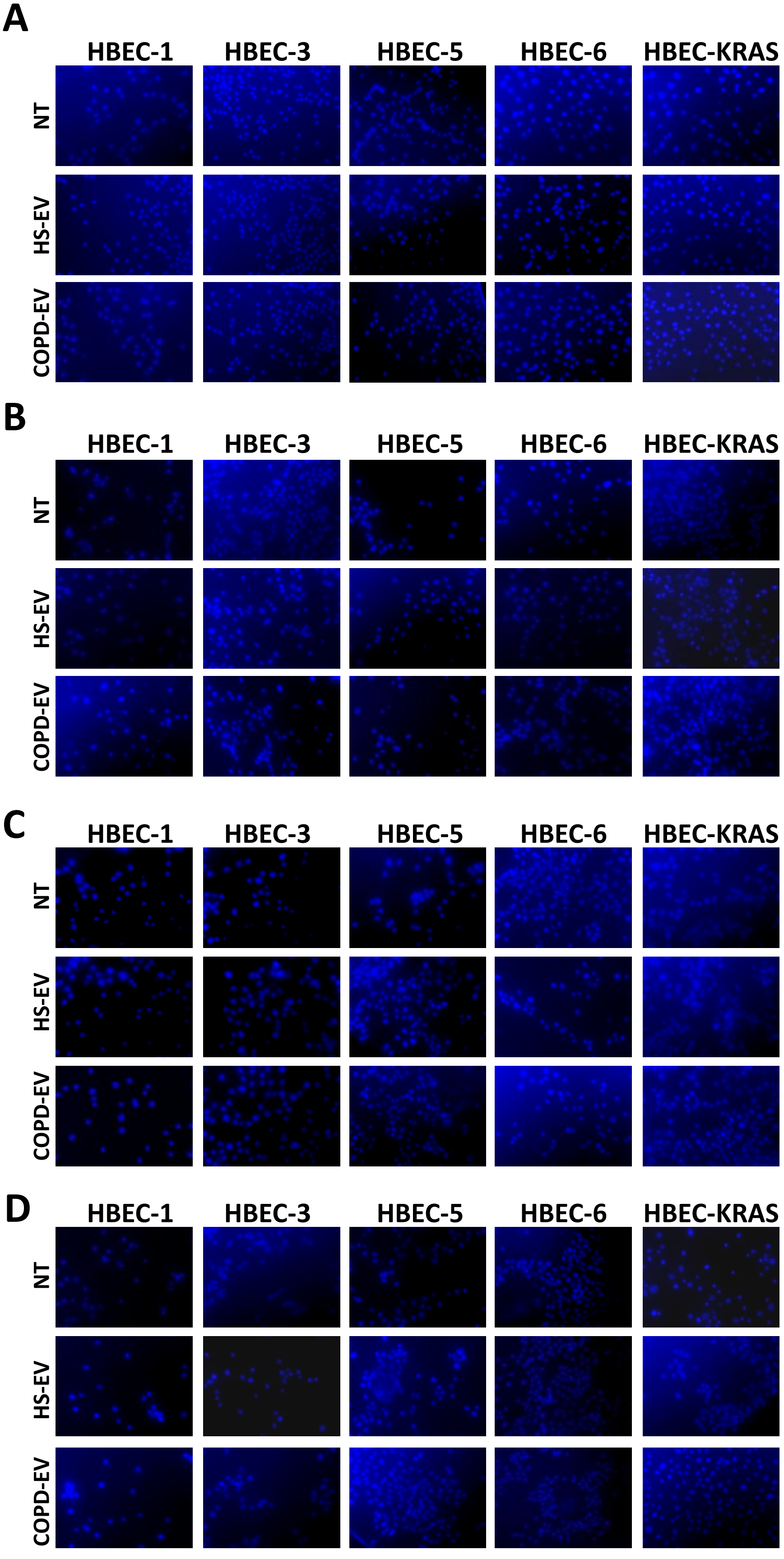

**Supplemental Figure 2**

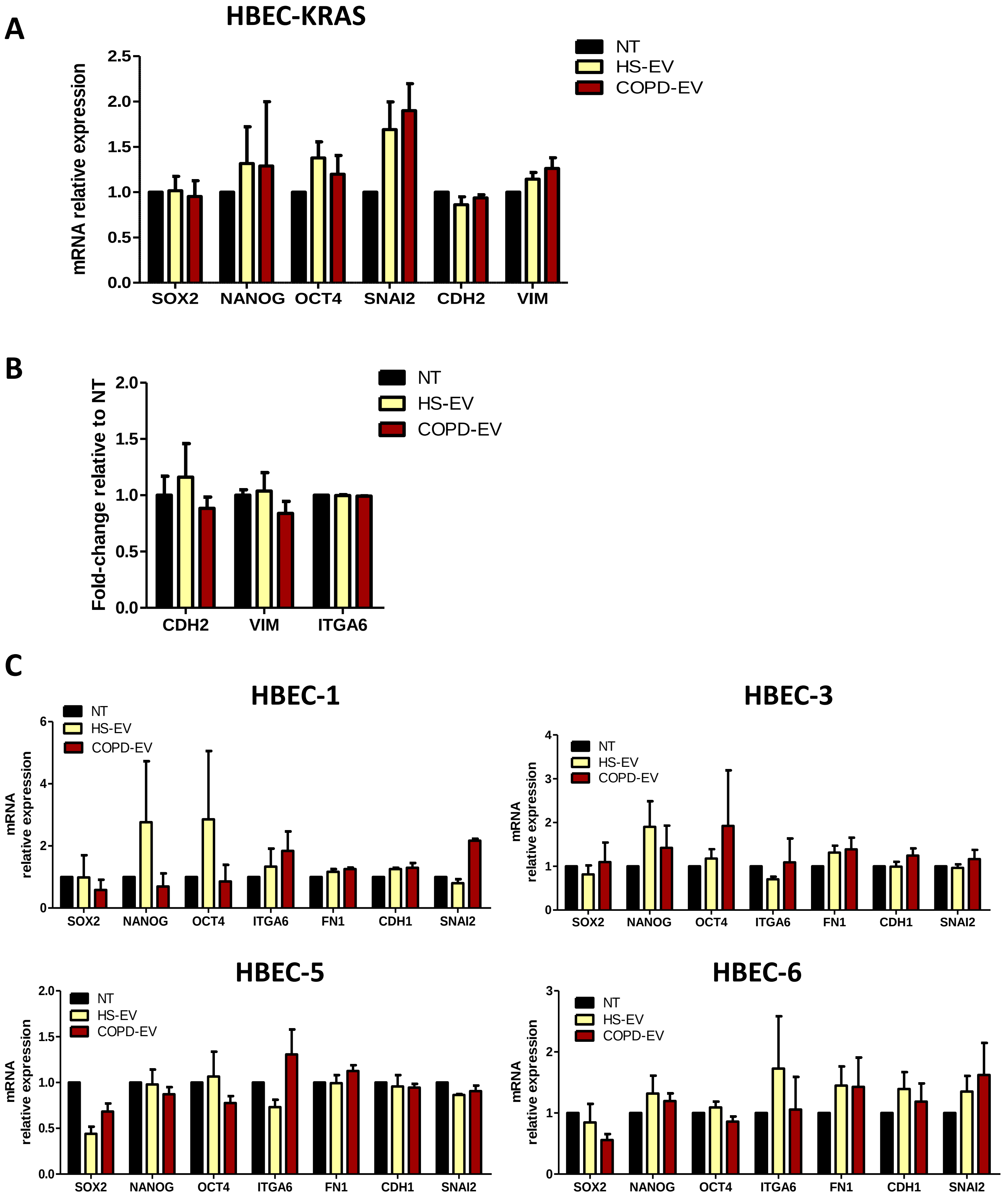

Supplemental Figure 3

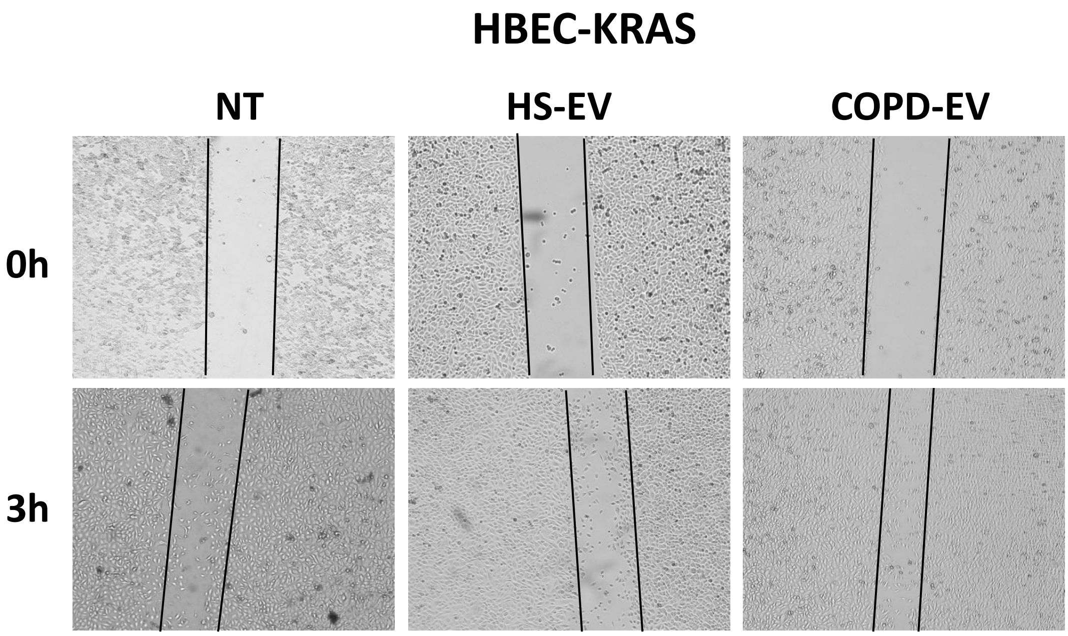

Supplemental Figure 4

A

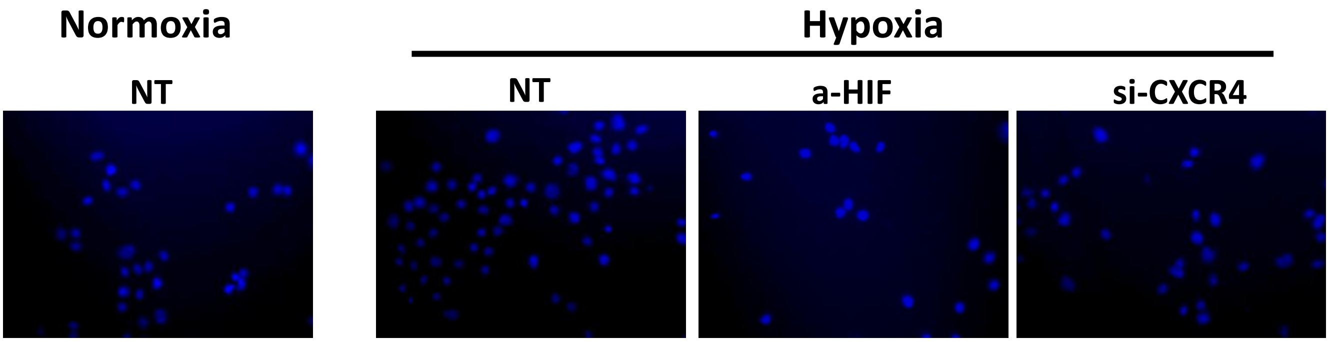


B

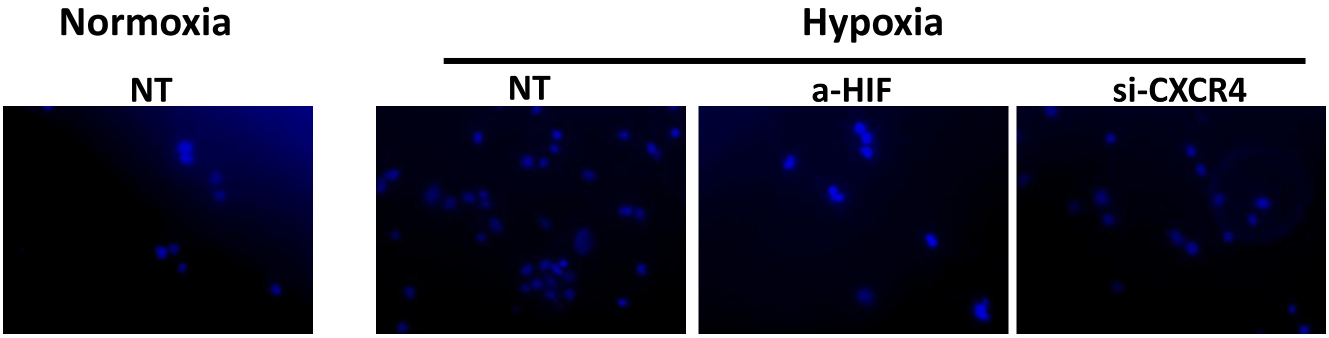

## Supplemental Figure 5

**A**

## CD9

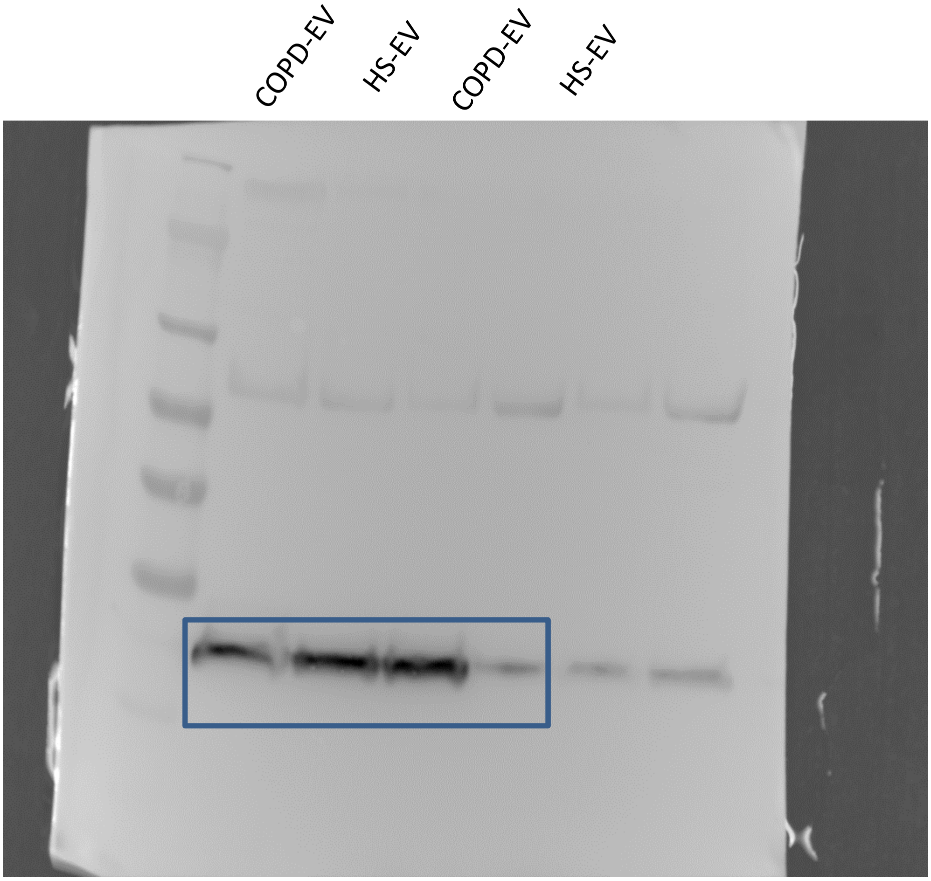


**B**

## CD81

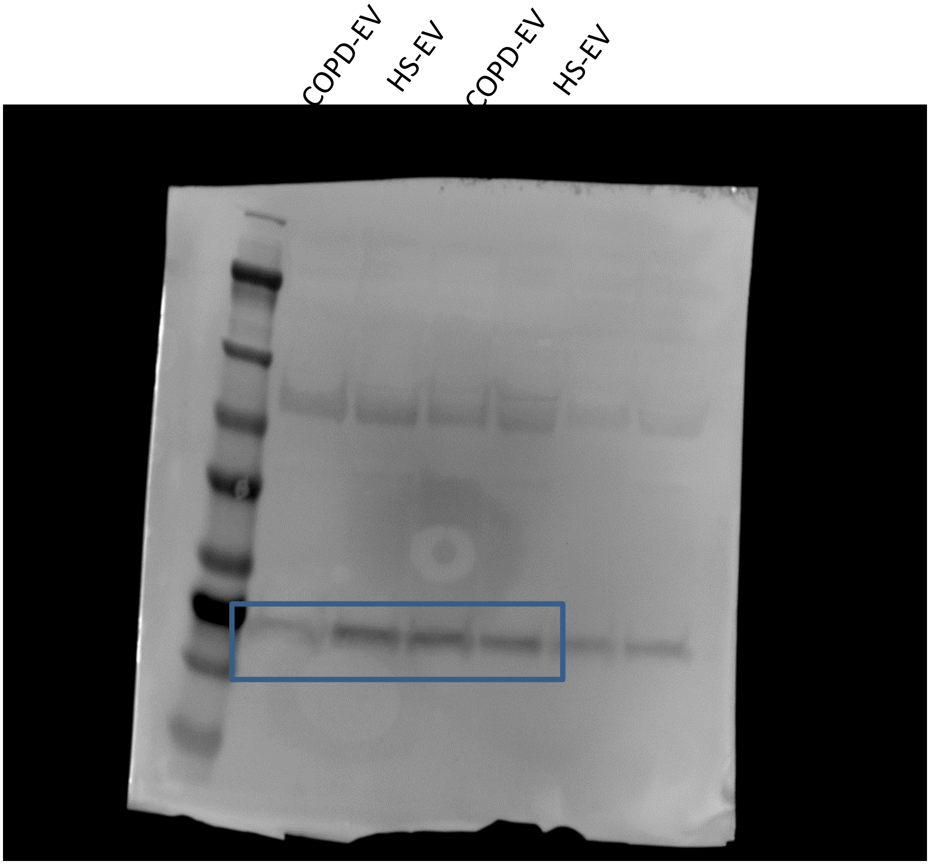

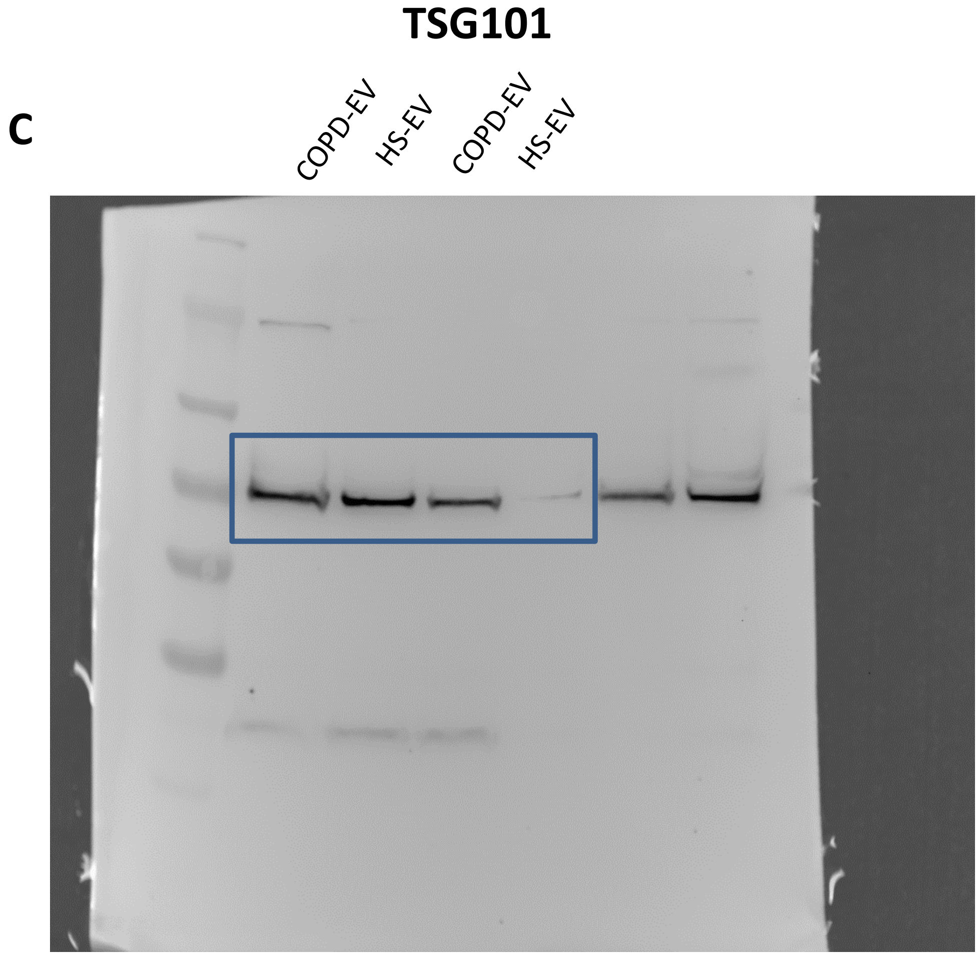
TSG101
C
COPD-EV
HS-EV
COPD-EV
HS-EV

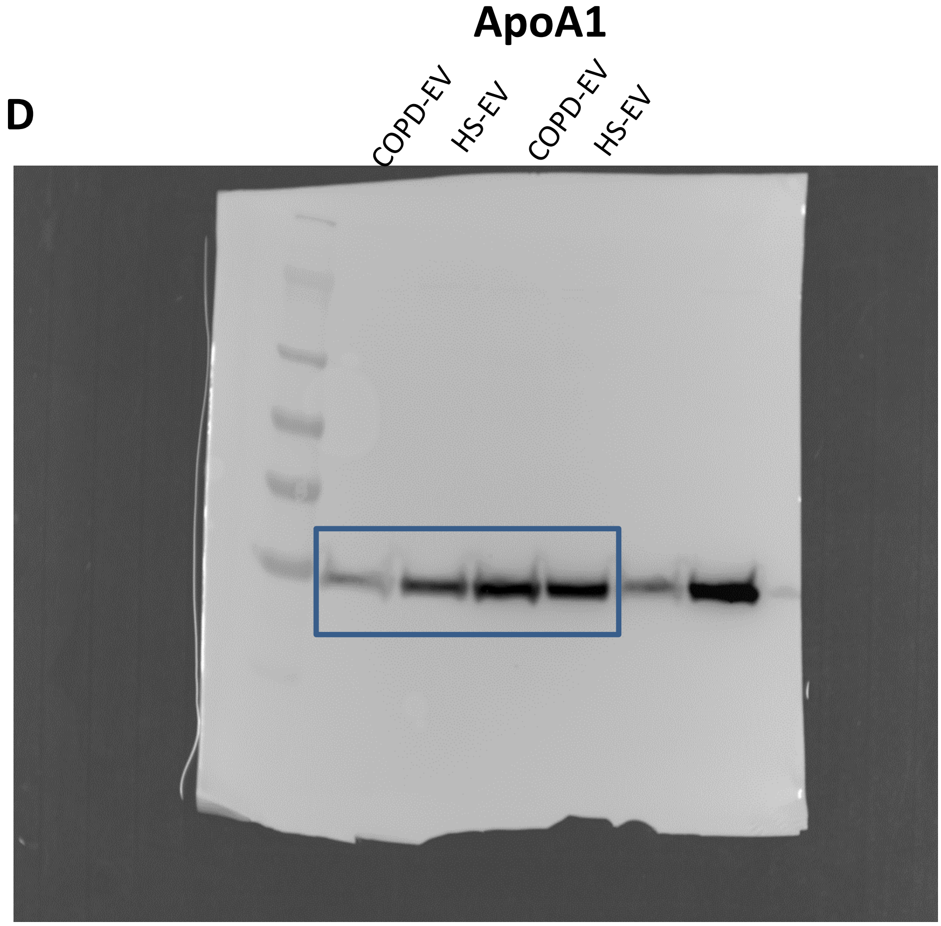
ApoA1
D
COPD-EV
HS-EV
COPD-EV
HS-EV
